# Epigenetic Findings in Autism: New Perspectives for Therapy

**DOI:** 10.3390/ijerph10094261

**Published:** 2013-09-11

**Authors:** Dario Siniscalco, Alessandra Cirillo, James Jeffrey Bradstreet, Nicola Antonucci

**Affiliations:** 1 Department of Experimental Medicine, Second University of Naples; via S. Maria di Costantinopoli, Napoli 16-80138, Italy; 2 Centre for Autism—La Forza del Silenzio, Caserta 81036, Italy; 3 Cancellautismo—Non-Profit Association for Autism Care, Florence 50132, Italy; 4 Institute of Protein Biochemistry, National Research Council of Italy; Naples 80128, Italy; E-Mail: cirillo.alessandra@libero.it; 5 International Child Development Resource Center, Chateau Elan 30517, USA; E-Mail: drbradstreet@gmail.com; 6 Biomedical Centre for Autism Research and Treatment, Bari 70126, Italy; E-Mail: info@antonucci.eu

**Keywords:** autism, gene expression, environmental factors

## Abstract

Autism and autism spectrum disorders (ASDs) are complex neurodevelopmental disorders characterized by dysfunctions in social interactions, communications, restricted interests, and repetitive stereotypic behaviors. Despite extensive genetic and biological research, significant controversy surrounds our understanding of the specific mechanisms of their pathogenesis. However, accumulating evidence points to the involvement of epigenetic modifications as foundational in creating ASD pathophysiology. Epigenetic modifications or the alteration of DNA transcription via variations in DNA methylation and histone modifications but without alterations in the DNA sequence, affect gene regulation. These alterations in gene expression, obtained through DNA methylation and/or histone modifications, result from transcriptional regulatory influences of environmental factors, such as nutritional deficiencies, various toxicants, immunological effects, and pharmaceuticals. As such these effects are epigenetic regulators which determine the final biochemistry and physiology of the individual. In contrast to psychopharmacological interventions, bettering our understanding of how these gene-environmental interactions create autistic symptoms should facilitate the development of therapeutic targeting of gene expression for ASD biomedical care.

## 1. Autism

Autism spectrum disorders (ASDs) represent complex, pervasive neuro-developmental disabilities [1], characterized by dysfunctions in social interactions, communications and restricted/fixated interests or repetitive behavior that manifest in early childhood [2,3]. Most children with ASDs (about 50%–70%) are intellectually disabled by nonverbal IQ testing, and are at significant risk of developing seizures [4]. Autism is a lifelong disability requiring intensive parental, school, and social support, rendering autism an urgent health care priority.

Currently, there is no definitive pharmacotherapy for the treatment of core symptoms of ASDs [3]. Therapies commonly used for ASDs involves educational, behavioral, sensory-based, nutritional, hyperbaric oxygen, heavy metal detoxification, immunological interventions, and a variety of symptom-directed pharmacological approaches [5,6,7,8]. These pharmacological options (*i.e*., atypical antipsychotic drugs) usually only target the secondary symptoms, such as aggression, irritability, depression, anxiety and self-injurious behaviors. Dietary and behavioral therapy may help improve language, social interactions and communication skills.

While the etiopathogenesis of ASDs remains elusive and controversial, it is now well recognized that ASDs involve the complex interaction of several genes and environmental risk factors [9,10,11]. As ASDs result from a complex combination of genetic, epigenetic, environmental (*i.e*., infections, toxins, air pollution, organophosphates, heavy metals, stressors), and immunological factors, these pathologies could be referred as multifactorial and polygenic disorders [12,13,14]. It seems likely that epigenetic dysregulation might contribute to significant proportion of ASD cases [15]. Even if specific chromosomal regions have been identified in autism-susceptibility loci, the results have been inconclusive, and the identification of the underlying genes has failed to produce a substantial causal linkage [16]. No single gene can account for more than 1% of the cases of ASDs.

## 2. Epigenetics

The term epigenetics was first coined in the 1940s by British embryologist and geneticist Conrad Waddington, who described it as: “the interactions of genes with their environment, which bring the phenotype into being” [17]. Our present knowledge enhances this earlier understanding, and epigenetics now evaluates the alteration of DNA transcription via variations in DNA methylation and histone modifications, but without alterations in the DNA sequence. These variants represent the epigenome, which in turn will be reflected in the transcriptome: that portion of the DNA which is being actively transcribed into RNA. However, it is noteworthy to consider that the non-coding RNA represents over 90% of the transcripts in most cells. Regions of DNA that do not code for proteins (*i.e*., intergenic regions) can be actively transcribed and participate in the genes regulation, and is known as non-coding RNA. Epigenetic factors can impact large-scale “omics”-type cellular processes: transcriptome, RNAome, proteome and metabalome [18,19]. 

### 2.1. DNA Methylation

In post-replication events, the DNA methyltransferase (DNA MTase) enzyme catalyzes the addition of a methyl group (CH_3_-) from the methyl donor *S*-adenosyl-l-methionine to the cytosine or adenine DNA nucleotides, typically at the C5 position of CpG dinucleotides. This biochemical process regulates the gene transcription and expression.

### 2.2. Histone Modifications

In mammalian cells, the basic unit of DNA packaging is the nucleosome. Histone basic proteins (histones) form the core around which the DNA is wrapped. This formation constitutes the chromatin [20]. The covalent modifications of the core histone proteins influence DNA availability to transcription processes, regulating in this way high-order DNA structure and gene expression.

## 3. Autism and Epigenetics

Since survival of any organism requires its ability to adapt to the various environmental factors it isn’t surprising to observe epigenetic influences more commonly than alteration of the DNA sequence [21]. The covalent modifications of DNA likely represent an interface between the changing environment and the fixed genome. Many environmental factors that have epidemiological association with ASDs exert their effects through epigenetic alterations [22]. Indeed, environmental factors can influence physiological process within cells, tissues and organs via changes in gene regulation. In example, endocrine-disrupting compounds (EDC) could affect ASD development [22]. Under this EDC category are included chemicals that affect endocrine glands, their function, hormone, receptors and signaling pathways. They are naturally occurring compounds (genistein), and/or synthetic compounds, such as the plasticizing agent bisphenol A (BPA), fluorosurfactants (perfluoro- octanesulfonic acid and perfluorooctanoic acid), herbicides (atrazine) and phthalate plasticizers [bis-[2-ethylhexyl] phthalate or di-2-ethylhexyl phthalate (DEHP)]; other compounds like lead, arsenic, dioxins, benzene, toluene are also included. Human exposure is almost unavoidable as these compounds are widespread in the environment (drinking water, household dust, several consumer products, like food and beverage containers, to name but a few possible sources). This point of view opens us to new insights into the environmental contributions to ASDs. With this in mind, new therapeutic approaches can be designed to address the consequences of the epigenetic influences.

## 4. DNA Methylation and Autism

A recent study has found a specific methylation pattern associated with ASD severity [23]. In addition, significant correlations between DNA methylation and quantitatively measured autistic trait scores were also identified, suggesting quantitative relationship between the severity of the autistic phenotype and epigenetic variation at several multiple loci previously implicated in the pathogenesis of ASDs, including *AFF2*, *AUTS2*, *GABRB3*, *NLGN3*, *NRXN1*, *SLC6A4* and *UBE3A*. However, systemic changes in epigenetic programming were not related to ASDs, whereas considerable variability was found in DNA methylation at individual CpG sites within ASD-discordant monozygotic twin pairs.

### Methylation Level of Specific Genes in ASDs

In ASDs, DNA methylation has been also linked to reduced expression of the oxytocin receptor [24]. Oxytocin is a neuropeptide hormone correlated with social behaviors. A connection between oxytocin and ASDs has been demonstrated [25]. Indeed, in a mouse model, the oxytocin receptor gene expression is epigenetically regulated by DNA methylation of its promoter [24]. Furthermore, methylation of specific sites in the gene promoter of the oxytocin receptor gene significantly inhibits its transcription in individuals with autism [24]. These findings also lead to an important question: are the social processes under epigenetic control? [26]. 

Genes that control synaptic molecules also show epigenetic regulation. Among them, SHANK3 gene is subjected to a specific epigenetic control mechanism [27]. Indeed, DNA methylation regulates the tissue-specific expression of SHANK3 gene [28]. Even if there are no studies indicating a direct association between the methylation status of Shank3 and ASD development, epigenetic dysregulation of the Shank-mediated connections could probably result in ASD pathogenesis. This gene comprises five CpG-islands, and it is associated with autism [29]. Specifically, the methylation of CpG-island 2 seems to be involved in the tissue-regulated expression. Shank3 mutant mice exhibit impaired social interaction and repetitive behaviors like autism. In the central nervous system, the protein SHANK3 is mainly expressed in neurons, especially in their synapses, and is strictly associated with the cell adhesion proteins neuroligins (NLGN), acting as a scaffolding protein. Neuroligins are a family of cell adhesion proteins located on the post-synaptic membrane. NLGNs are involved in the formation and maintainence of synapses between neurons. Neuroligins function is disrupted in ASDs, as specific mutations in the genes encoding NLGN 3 and NLGN 4 have been found, interfering with synaptic transmission [30]. In addition, it has been demonstrated that neuroligin-3 mutations alter tonic endocannabinoid signaling [31], providing evidence that alterations in the endocannabinoid pathway may contribute to autism pathophysiology. Recently, it has been found that cannabinoid type-2 (CB2) receptor is up-regulated in ASD- peripheral blood mononuclear cells (PBMCs) [32]. 

DNA methylation dysregulation also contributes to another key event in ASDs. Indeed, DNA methylation and pro-oxidant environmental stressors could modulate autism development. A link between epigenetic regulation and antioxidant/detoxification capacity has been reported in many children with autism that showed genome-wide DNA hypomethylation and oxidative protein/DNA damage [33]. Deficit in antioxidant and methylation capacity could promote cellular damage and altered epigenetic gene expression. 

Hyper-methylation of specific CpG sites in upstream promoter regions of BCL-2 and retinoic acid receptor-related orphan receptor (RORA) genes were identified in autistic children compared to healthy developing twins [34]. The expression of these genes was found down-regulated in the cerebellum of post-mortem brain tissues from subjects with ASDs. Bcl-2 is a known protein involved in the anti-apoptotic process [35]. These findings suggest that a possible dysfunctional apoptotic event could lead to altered development of specific brain regions, resulting in decreased cognitive function. RORA belongs to the nuclear receptor-1 subfamily of nuclear hormone receptors and is involved in the control of the neuronal oxidative stress [36]. RORA is a novel candidate gene for ASD pathology and could be related to sex hormones involvement in autism [37,38].

## 5. Histone Modifications and Autism

Neurodevelopmental pathologies, including ASDs, are affected by histone modifications [39,40]. Lysine acetylation, methylation, SUMOylation, and ubiquitinylation; arginine methylation; serine phosphorylation; proline isomerization are the covalent modifications of histone proteins. Most of them are localized to the amino- and carboxy-terminal histone tails, and a few are localized to the histone globular domains [41]. Lysine methylation of histone H3 could be involved in autism development [42]. The amino-acid lysine can carry up to three methyl groups. Each methylation could represent a distinct functional state of the cell [43]. Histone methylation machinery is involved in brain function and development. 

Alterations in this process could be related to autism. In pre-frontal cortex of autistics it has been identified altered methylation of H3K4 sequences in genes and loci implicated in regulating neuronal connectivity, social behaviors, and cognition [44]. Mutations in the X-linked gene SMCX which encodes a histone 3 lysine 4 (H3K4)me3-specific demethylase have been demonstrated [45]. This gene regulates in turn other genes, *i.e*., SCN2A, CACNA1H, BDNF, SLC18A1, associated with autism and cognitive dysfunction. Interestingly, it has been demonstrated a possible connection between epigenetic changes in ASD relevant behaviors and gene expression alterations. Indeed, histone deacetylase inhibitors sodium butyrate and trichostatin A were able to increase up-regulation of oxytocin receptor and vasopressin V1a receptor [46]. The genes encoding for these two receptors are strongly associated to ASD-like behaviors.

## 6. Environmental Factors Linked to Epigenetic Mechanisms in Autism

ASDs are now recognized as pathologies caused from several environmental risk factors [47]. While the genetics of autism are still incompletely elucidated, epigenetic mechanisms likely are the interface between the individual’s genetics and susceptibility to the environmental influences which subsequently develop the autistic phenotypic expression. Indeed, epigenetic modifications are known to be influenced by nutritional status, medications and even mental stress. A positive association between the U.S. Environmental Protection Agency (EPA)’s risk of neurological disease index, which was based on 23 air emission parameters, and autism in several US counties was found [48]. Another recent study found a positive correlation between exposure to traffic-related air pollution, nitrogen dioxide, PM_2.5_, and PM_10_ during pregnancy and during the first year of life with autism [49]. Prenatal exposure to anticonvulsant medication is a risk factor for the development of ASDs [50]. In animal models, it has been demonstrated that prenatal exposure to bisphenol A (BPA) affects mRNA levels of several genes encoded for estrogen receptors, oxytocin, and vasopressin, acknowledged as important neuropeptides modulators of diverse social behaviours (affiliative behavior—pair-bonding and maternal behavior), social cognition (social memory), and social approach (social preference or social avoidance)) [51,52,53]. Indeed, estrogen receptors, oxytocin, and vasopressin gene expressions variations are associated with differences in social interaction [54] and are strictly connected with ASDs, as dysregulations in these brain neuropeptide systems could underlie social dysfunctions in ASDs [24,55,56].

Polybrominated diphenyl ethers (PBDEs) exposure affects several proteins involved in neuronal survival, growth, and synaptogenesis, such as Brain-Derived Neurotrophic Factor (BDNF), Calcium-Calmodulin Kinase II (CaMKII) and growth-associated protein-43 (GAP-43) [57], indicating that the exposure to several environmental chemicals can be a candidate factor associated with the development of ASDs [58]. Indeed, these proteins are involved in normal brain maturation. Clinical data and animal models indicate that they are dysregulated in ASDs. Altered BDNF levels have been found in autistic patients [59]. BDNF is a neurotrophic factor and important regulator of neuronal functions [60]. Several evidences indicate an involvement of BDNF in ASDs [61]. Interestingly, BDNF plays a role as trophic support for serotonergic neurons, and serotonin levels are altered in ASDs [59]. 

Reduction in the phosphorylation of CAMKII was found in the maternal immune activation (MIA) autism mouse model of gestational poly(IC) exposure [62]. Overexpression of GAP-43 is linked to excessive number of thin axons in cortex areas of autistic subjects [63].

It has been recently proposed that environmental factors-associated epigenetic changes in epitestosterone synthesis could contribute to the development of autistic-like behaviors in rodents administered with a postnatal dose of citalopram, estradiol or valproic acid [64]. Dietary vitamin D could regulate epigenetic events. Maternal vitamin D deficiency has been indicated as a risk factor for the development of infantile autism [65,66]. Vitamin D and its receptor (VDR) are involved in the regulation of several genes controlling inflammation, immunity, cellular proliferation, differentiation, and apoptosis. Indeed, nuclear VDR activated by a metabolite of vitamin D, the 1,25-dihydroxyvitamin D(3), cooperates with some chromatin modification enzymes (*i.e*., histone acetyltransferases and histone deacetylases), taking a role in complex epigenetic events [67]. 

The influence of endogenous and exogenous factors on genotype could impact the metabolic phenotype. It has been demonstrated that autistic patients show differences in allele frequency and/or significant gene-gene interactions for relevant genes encoding for the protein involved in methionine metabolism, such as the reduced folate carrier, transcobalamin II, catechol-o-methyltransferase, methylenetetrahydrofolate reductase, and glutathione-S-transferase, indicating an increased vulnerability to oxidative stress (endogenous or environmental) [68].

## 7. Other Process

Beyond the DNA methylation changes, novel findings indicate RNA methylation changes could have functional implications, *i.e*., on mRNA life-cycle, and could be related to glutathione anti-oxidant levels [69]. Indeed, this methylation could be responsible to regulate mRNA maturation at both pre-transcription level, by regulating precursor-RNA processing and splicing into mRNA, and post-transcription level by regulating the functions of ribonucleoproteins and RNA binding proteins in mRNA translation. The methylation process is under the control of glutathione anti-oxidant levels, indicating that the redox status of neurons could act as central regulatory switch for methylation-based changes in mRNA processing. This aspect could be very interesting in ASDs, since these syndromes show strong imbalance in glutathione levels [70], enhancing the redox/methylation hypothesis of autism [71]. According to this theory, the oxidative stress, mediated by environment factors, could trigger impaired methylation and, consequently, neurological deficits.

## 8. Autistic-Like Syndromes

Beyond ASDs, other autism-like disorders show epigenetic regulation. However, differently from autism, these diseases are clinically recognizable genetic syndromes. Rett syndrome (RTT), a disease caused by mutations in the gene coding for methyl CpG-binding protein 2 (MeCP2), is associated to defects in epigenetic modifiers [72]. RTT patients show alteration of the chromatin state of MeCP2 target genes: increase in the density of histone H3 and decreased levels of trimethylation of lysine 4 on histone H3 (H3K4me3), a modification associated with transcriptional activation [73].

Fragile X syndrome (FXS) is a monogenetic disease that causes intellectual disability and autism-like behavior [74]. The FXS mutation is an huge expansion of a trinucleotide repeat in the 5′ untranslated region (UTR) of the X-linked gene fragile X mental retardation 1 gene (FMR1) [75]. These polymorphic CGG triplet repeats (>200 CGG) in the 5′UTR of FMR1 gene are able to trigger heterochromatin formation, histone deacetylation and trimethylation at critical residues H3K9 and H3K27, and DNA methylation across the promoter and through the repeat. Heterochromatin formation and DNA methylation silence FMR1 transcription [76]. It has been demonstrated that FXS cells show a decrease in methylation of histone H3 at lysine 4 together with a large increase in methylation at lysine 9. These events could switch from euchromatin to heterochromatin in the disease state [77].

The most consistent known chromosomal abnormality reported that often includes autism is the duplication of the 15q11-q13 segment, which contains a cluster of imprinted key genes essential for normal neurodevelopment [78]. The duplications of chromosome 15q11-q13 are the only commonly recurrent cytogenetic aberration associated with ASDs [79] and reveals epigenetic alterations in gene expression [80]. Among the genes present in the 15q11-q13 segment, there is the cluster of three GABA(A) receptor subunit (GABR) genes; some autistic patients and RTT patients show common epigenetic dysregulation of these genes [81].

## 9. Conclusions and Perspectives

Although the role played by epigenetics in ASDs remains in its infancy, as well as the exact epigenetic account for the ASDs cases, these data infer that ASDs may be “epigenopathies”, and further research will be needed to fully characterize epigenetic mechanisms in ASDs. DNA methylation and histone modifications can be analyzed through technological advances, *i.e*., combining chromatin immunoprecipitation with single DNA molecule sequencing [82], providing detailed epigenetic information. Understanding the epigenetic processes in ASDs is essential for developing targeted epigenetic therapy. Indeed, epigenetic mechanisms can be modifed. Epigenetic drugs targeting DNA methylation and histone deacetylation enzymes are able to reverse abnormal gene expression profiles [83], offering new tools to control the development of complex disorders like autism. This opens a new era of patient-specific therapies for children with ASDs. Epigenetic drugs, such as DNA methyltransferases inhibitors azacitidine and decitabine, have been mostly investigated as anti-tumor drugs. However, they also show other properties, such as immunomodulatory effects [84]. Low doses of these drugs could be helpful in ASD therapy. Histone deacetylase inhibiting agents could be used as neuroprotective drugs [85]. It is noteworthy to consider that currently no studies or clinical trials with epigenetic drugs have been conducting for ASD treatment.
